# Congestive Heart Failure and Arrhythmias Among Hospitalized Patients With Carcinoid Syndrome

**DOI:** 10.7759/cureus.81958

**Published:** 2025-04-09

**Authors:** Etuk Aniekeme, Bruno Goncalves, Sneha Pillai, Demilade Soji-Ayoade, Komal Sodhi, Carlos Rueda Rios, Ellen Thompson

**Affiliations:** 1 Department of Cardiology, Marshall University Joan C. Edwards School of Medicine, Huntington, USA; 2 Department of Biomedical Sciences, Marshall University Joan C. Edwards School of Medicine, Huntington, USA; 3 Department of Surgery, Marshall University Joan C. Edwards School of Medicine, Huntington, USA

**Keywords:** arrhythmia, carcinoid syndrome, congestive heart failure, healthcare, predictors

## Abstract

Introduction

Carcinoid syndrome (CS) represents the most common functional syndrome in patients with neuroendocrine tumors which may be in an advanced tumor state. The pathophysiology of congestive heart failure (CHF) and arrhythmia in CS is poorly understood; however, chronic exposure to excessive circulating serotonin is considered one of the most important factors contributing to increased morbidity and mortality in this population. Despite recognition, international consensus guidelines specifically addressing the diagnosis and management of CHF and arrhythmia in CS are lacking. This study focused on hospitalized patients once they represent more severe disease states requiring intensive management, thereby providing a clearer understanding of factors influencing adverse clinical outcomes.

Methods

This retrospective cohort study utilized the Healthcare Cost and Utilization Project National Inpatient Sample to identify predictors of congestive heart failure and arrhythmia in hospitalized CS patients from 2016 to 2018.

Results

Initially, a total of 1,859 patients were included. After stratification, 606 patients had CHF and arrhythmia diagnosis with variables analyzed using multivariate logistic regression. Among 606 patients, 360 had CHF with advanced age, male sex, non-Hispanic Black race, Medicare insurance, and prolonged hospital stays, all of which were identified as significant predictors. Similarly, 246 patients diagnosed with arrhythmias were more prevalent in older and male patients and were associated with increased mortality and prolonged hospitalization.

Conclusion

These results highlight critical patient-related factors influencing mortality in CS patients with CHF and arrhythmia. Strategies aimed at early recognition, including clinical scoring systems, and biomarker assessment, could improve risk stratification and patient outcomes. Our findings underscore the importance of early risk stratification and targeted interventions to mitigate cardiac complications in CS patients. Additionally, incorporating our data into clinical practice has the potential to improve early recognition, promoting timely interventions for CS patients with CFH and arrhythmia. Developing consensus guidelines for managing CS-related cardiac complications, based on these insights, will further standardize care and improve patient outcomes.

## Introduction

Carcinoid syndrome (CS) represents the most common functional syndrome with a reported prevalence of approximately 19% in the neuroendocrine tumors (NETs) patients’ population [1]. NETs are rare malignancies that can develop from a diffuse network of neuroendocrine cells throughout the body. The incidence is a five-fold increase with an annual incidence of 5·25/100,000 in the United States in the last decade [2]. This five-fold increase refers specifically to NETs, while the 19% prevalence indicates that nearly one in five NET patients develop CS, underscoring the clinical relevance of this association. The massive release of tumor-derived bioactive peptides, such as serotonin, into the systemic circulation induces the clinical picture [3]. Excessive amounts of circulating serotonin and other substances produce a wide range of symptoms, commonly manifesting as increased bowel movements and bronchoconstriction, and may result in fibrotic complications in heart structure [4].

Approximately 20-40% of patients with CS develop cardiac involvement, including carcinoid heart disease (CHD), and congestive heart failure (CHF), which are of paramount clinical importance. While CHF often develops secondary to CHD due to volume overload and valvular dysfunction, it can also occur independently in CS patients without CHD, demonstrating the diverse cardiac manifestations of CS. Although the pathophysiology behind CS and cardiac dysfunction is not clearly defined, the majority of relevant publications attribute its core mechanism to chronic overexposure to serotonin in cardiac cells [5]. Serotonin and other bioactive compounds such as neuropeptide K and transforming growth factor-β released by the tumor can cause mitosis in fibroblasts and smooth muscle cells and induce endocardium fibrosis [6-9]. Also, serotonin contributes to plaque deposition on various endocardial surfaces such as the valve leaflets, sub-valvular apparatus, and cardiac chambers, commonly affecting the right-side heart valves, leading to tricuspid regurgitation and pulmonary stenosis [10, 11]. The resulting volume overload and progressive cardiac dysfunction contribute to CHF [12]. In addition, to promoting valvular dysfunction and myocardial fibrosis, serotonin directly affects cardiac contractility by modulating intracellular calcium levels and altering β-adrenergic receptor sensitivity, which may exacerbate systolic and diastolic dysfunction [13-16].

Arrhythmia is observed in approximately 18% of carcinoid patients [16], although the precise underlying pathophysiological mechanisms remain incompletely understood. Serotonin has arrhythmogenic properties and may have the main role in the development of arrhythmia in CHD patients [17, 18]. Serotonin-induced myocardial fibrosis alters the atrium from valvular dysfunction, promoting calcium release. Dysregulation of calcium homeostasis, mediated by serotonin’s effects on L-type calcium channels and sarcoplasmic reticulum function, leads to intracellular calcium overload, facilitating arrhythmogenesis, further intensifying cardiovascular complications [19-25]. Certain arrhythmia subtypes, particularly atrial fibrillation, and ventricular arrhythmia, are more frequently reported in CS patients, reflecting the proarrhythmic substrate created by serotonin-induced fibrosis and calcium overload [25, 26]. Moreover, chronic serotonin exposure is associated with endothelial dysfunction and increased oxidative stress, exacerbating myocardial inflammation and electrical instability [27-29]. Also, oxidative stress disrupts the redox balance within cardiomyocytes, leading to increased reactive oxygen species (ROS) production, which interferes with ion channel function and gap junction integrity, further contributing to arrhythmic vulnerability [30].

The prognosis of patients with CHD treated medically is poor, with a three-year survival rate as low as 31% [31]. This survival rate primarily reflects outcomes in medically managed patients. In comparison, CS patients without CHD have a more favorable prognosis, reinforcing the detrimental impact of cardiac involvement on survival outcomes. This study, therefore, aims to identify key predictors of CHF and arrhythmias among hospitalized patients with CS including demographic factors and clinical characteristics to establish a comprehensive risk profile for CS-related cardiac complications. By understanding these associations, we can provide valuable insights into the clinical implications of CHF and arrhythmia in CS patients and potentially guide healthcare providers in implementing target strategies for early risk stratification and management improving clinical outcomes and reducing mortality in CS patients with cardiac involvement.

## Materials and methods

Patient selection and data collection

This study was conducted as a retrospective cohort study utilizing data from the Healthcare Cost and Utilization Project National Inpatient Sample (HCUP-NIS). This dataset reflects approximately 97% of the US population, referring to all inpatient hospitalizations at non-federal, short-term, general, and specialty hospitals, facilitating the derivation of national estimates concerning hospital utilization [32]. The database is constructed using a stratified sampling methodology, capturing 20% of discharges from participating institutions, thereby ensuring its applicability to broader population analyses. For the identification of eligible study participants, trained clinicians analyzed HCUP-NIS records covering the period from 2016 to 2018. Selection criteria were based on the International Classification of Diseases, Tenth Revision, and Clinical Modification (ICD-10-CM) codes specific to CS. The inclusion criteria required the selection of all hospitalizations for CS in individuals aged ≥18 years, confirmed by codes. In addition, the exclusion criteria encompassed patients under 18 years of age and patients without CHF and arrhythmia. Relevant clinical and demographic data were collected for individuals matching the eligibility criteria. Due to the small sample size for Hispanic participants (n = 10), they were combined with other racial/ethnic groups (Asian or Pacific Islander, Native American, and other categories as defined by the NIS data element) for analysis.

Statistical analysis

Appropriate weights were applied to the data to account for the complex survey design and make them representative of the general population. The chi-square test was used to compare the categorical variables between patients with and without CHF as well as with and without arrhythmia, and multivariate logistic was performed to analyze the association. All statistical analyses were performed using Stata SE 16.1 (Stata Corp, College Station, TX). Statistical significance was determined at a threshold of p<0.05, and all comparative assessments were conducted accordingly.

## Results

In this study, we analyzed sociodemographic, hospital-level, and clinical characteristics of hospitalized patients with CS to identify predictors of CHF and arrhythmias. A total of 1,859 patients had CS and were included in the study. After the first stratification, 360 patients, approximately 19.4%, had a CHF diagnosis. Table 1 summarizes the basic characteristics of the study population. Our data showed that patients with CHF were significantly older, with 64.17% being 65 years or more, compared to the non-CHF group. Additionally, males had a higher prevalence of CHF, with 49.44% compared to female patients. Regarding racial/ethnic distribution, non-Hispanic Black patients had a significantly higher proportion of CHF. Our results demonstrated that patients with CHF were more likely to have Medicare insurance. We also analyzed the mortality rates in this population. Our findings showed that the CHF group had a significantly higher mortality rate as well as a longer hospital stay (>5 days) compared to the non-CHF group. In addition, a higher Charlson comorbidity index (CCI) was observed among CHF patients, with 19.44% having a CCI≥ 8 compared to the non-CHF group. However, hospital region, hospital location, and median household income were not significantly associated with CHF.

**Table 1:** Sociodemographic, Hospital-Level, and Clinical Characteristics of Hospitalized Patients With Carcinoid Syndrome by Heart Failure Diagnosis. N=unweighted number of observations; WT%=weighted percentages. Race/ethnicity categories follow the NIS database classification. "Non-Hispanic other" includes Asian or Pacific Islander, Native American, and other racial groups per NIS definitions. Asian or Pacific Islander, Native American and other racial individuals were included in the "Non-Hispanic other" category due to a sample size below the reporting threshold (n=10) per NIS database recommendations.

Characteristics	Congestive Heart Failure (WT %)		Likelihood Ratio Tests		
	Yes (n = 360)	No (n = 1499)	Chi-square	df	p-value
Age			14.6583	1	<0.001
Less than 65	35.83	46.96			
65 years and above	64.17	53.04			
Gender			4.8833	1	0.0273
Male	49.44	42.9			
Female	50.56	57.1			
Race/ethnicity			5.3433	3	0.0012
Non-Hispanic White	70.2	79.01			
Non-Hispanic Black	19.2	11.15			
Hispanic	4.3	5.85			
Non-Hispanic other	6.3	3.99			
Median household income national quartiles			1.3741	3	0.2491
Quartile 1	24.29	21.26			
Quartile 2	29.94	28.06			
Quartile 3	24.29	24.52			
Quartile 4	21.47	26.15			
Insurance type			11.6783	2	<0.001
Medicare	71.87	58.61			
Medicaid	5.29	7.54			
Private/Other	22.84	33.85			
Hospital region			1.4849	3	0.217
Northeast	14.72	17.75			
Midwest	28.89	24.88			
South	38.06	36.56			
West	18.33	20.81			
Hospital location			0.5178	2	0.596
Rural	6.11	7.4			
Urban nonteaching	19.44	19.88			
Urban teaching	74.44	72.72			
Mortality			5.8236	1	0.016
No	91.67	95.33			
Yes	8.33	4.67			
Admission type			12.1519	1	<0.001
Elective	13.97	21.09			
Non-elective	86.03	78.91			
Length of stay			9.4129	1	0.0022
5 days or less	51.11	60.04			
More than 5 days	48.89	39.96			
Charlson Comorbidity Index			26.2684	2	<0.001
0 – 4	50.56	72.25			
5 – 7	30	14.94			
⩾ 8	19.44	12.81			

Table 2 shows the factors associated with CHF among hospitalized patients with carcinoid syndrome. Our multivariate analysis revealed that age (≥65 years), which was significant in the unadjusted model (OR 1.58, 1.24-2.02), lost significance in the adjusted model (AOR 1.23, 0.89-1.69), suggesting that the effect of age may have been confounded by other factors such as comorbidities or medication use. Additionally, the non-Hispanic Black race (AOR 2.05, 1.43-2.94) and the non-Hispanic/other race (AOR 2.07, 1.18-3.65) were independent predictors of CHF. Private insurance was associated with a reduced risk of CHF (AOR 0.65, 0.46-0.93) compared to Medicare. This reduction in risk with private insurance may reflect better access to outpatient care, which could prevent CHF complications. We also demonstrated that increased hospital length of stay (>5 days) was associated with higher odds of CHF (AOR 1.35, 1.05-1.73). Patients with higher CCI scores also had greater odds of CHF, with an AOR of 2.61,1.97-3.46 for CCI 5-7 and 1.71 (1.21-2.43) for CCI ≥ 8. Additionally, mortality remained a significant predictor, with an AOR of 1.67 (1.05-2.66). Other factors, such as age, gender, median house income, hospital region, hospital location, and admission type, did not show an association with CHF. Admission type (elective vs. non-elective) was non-significant after adjustment (AOR 0.76, 0.55-1.05), possibly because CHF is often managed electively in early stages, but its progression can lead to non-elective admissions.

**Table 2:** Factors Associated With Congestive Heart Failure Among Hospitalized Patients With Carcinoid Syndrome OR = Odds ratio; CI = Confidence Interval, models adjusted for age, race/ethnicity, gender, hospital region, hospital location, Charlson comorbidity index, insurance, length of stay, mortality, type of admission, and Median Household income national quartiles. The “Non-Hispanic Other” category includes Asian or Pacific Islander, Native American, and other races per NIS database classification. Asian or Pacific Islander, Native American and other racial individuals were included in the "Non-Hispanic other" category due to a sample size below the reporting threshold (n=10) per NIS database recommendations. * Indicates statistical significance of p<0.05.

Characteristics	OR (95% CI)	
	Unadjusted	Adjusted
Age		
Less than 65	Reference	Reference
65 years and above	1.58 (1.24–2.02)*	1.23 (0.89–1.69)
Gender		
Male	Reference	Reference
Female	0.77 (0.61–0.97)*	0.79 (0.61–1.01)
Race/ethnicity		
Non-Hispanic White	Reference	Reference
Non-Hispanic Black	1.94 (1.41–2.67)*	2.05 (1.43–2.94)*
Hispanic	0.83 (0.47–1.44)	0.84 (0.44-1.59)
Non-Hispanic other	1.78 (0.99–3.20)	2.07 (1.18–3.65)*
Median household income national quartiles		
Quartile 1	Reference	Reference
Quartile 2	0.93 (0.68–1.29)	1.05 (0.73–1.50)
Quartile 3	0.87 (0.62–1.22)	0.96 (0.66–1.41)
Quartile 4	0.72 (0.51–1.01)	0.85 (0.57–1.27)
Insurance type		
Medicare	Reference	Reference
Medicaid	0.57 (0.34–0.96)*	0.54 (0.28–1.01)
Private/Other	0.55 (0.42–0.72)*	0.65 (0.46–0.93)*
Hospital region		
Northeast	Reference	Reference
Midwest	1.40 (0.97–2.01)	1.39 (0.93–2.06)
South	1.25 (0.88–1.79)	1.07 (0.73–1.58)
West	1.06 (0.71–1.59)	1.06 (0.68–1.65)
Hospital location		
Rural	Reference	Reference
Urban nonteaching	1.18 (0.72–1.95)	1.17 (0.66–2.06)
Urban teaching	1.24 (0.80–1.93)	1.24 (0.75–2.05)
Mortality		
No	Reference	Reference
Yes	1.85 (1.20–2.85)*	1.67 (1.05–2.66)*
Admission type		
Non-elective	Reference	Reference
Elective	0.61 (0.45–0.82)*	0.76 (0.55–1.05)
Length of stay		
5 days or less	Reference	Reference
More than 5 days	1.44 (1.14–1.81)*	1.35 (1.05–1.73)*
Charlson Comorbidity Index		
0 – 4	Reference	Reference
5 – 7	2.87 (2.18–3.78)*	2.61 (1.97–3.46)*
⩾ 8	2.17 (1.58–2.98)*	1.71 (1.21–2.43)*

After the stratification, 246 patients were included in the study with an arrhythmia diagnosis. Table 3 indicates that patients diagnosed with arrhythmia were significantly older, with 72.36% being 65 years or older compared to the non-arrhythmia group. Our data showed that there was a higher prevalence of male patients in the arrhythmia group compared to the non-arrhythmia group. For racial/ethnic distribution, no difference was found between groups, and no associations were observed with median household income, hospital region, hospital location, admission type, as well as Charlson Comorbidity Index. However, our results demonstrated that Medicare is the predominant payer among arrhythmia patients, while private insurance was more frequent in the non-arrhythmia group. This difference may reflect the older age distribution in the arrhythmia group, while patients with private insurance may have had better pre-hospitalization arrhythmia management, reducing the need for hospitalization. In addition, mortality was significantly higher in patients with arrhythmias, and they also had prolonged hospitalizations compared to the non-arrhythmia group. Although the association between mortality and arrhythmia (AOR 1.56, 0.90-2.70) did not reach statistical significance, the p-value was approaching significance, indicating a possible trend toward increased mortality in this group.

**Table 3:** Sociodemographic, Hospital-level, and Clinical Characteristics of Hospitalized Patients With Carcinoid Syndrome by Arrhythmia Diagnosis. N=unweighted number of observations; WT%=weighted percentages. Race/ethnicity categories follow the NIS database classification. "Other" includes Hispanics, Asian or Pacific Islander, Native American, and other racial groups per NIS definitions. Hispanic, Asian or Pacific Islander, Native American and other racial individuals were included in the "Other" category due to a sample size below the reporting threshold (n=10) per NIS database recommendations.

Characteristics	Arrhythmias (WT%)		Likelihood Ratio Tests		
	Yes (n = 246)	No (n = 1613)	Chi-Square	df	p-value
Age			35.3101	1	<0.001
Less than 65	27.64	47.43			
65 years and above	72.36	52.57			
Gender			14.6272	1	<0.001
Male	55.69	42.41			
Female	44.31	57.59			
Race/ethnicity			1.5842	2	0.2056
Non-Hispanic White	81.59	76.65			
Non-Hispanic Black	10.88	12.99			
Other	7.53	10.36			
Median household income national quartiles			0.8262	3	0.4795
Quartile 1	18.37	22.39			
Quartile 2	28.16	28.46			
Quartile 3	25.71	24.29			
Quartile 4	27.76	24.86			
Insurance type			9.8148	2	<0.001
Medicare	72.76	59.4			
Medicaid	6.91	7.14			
Private/Other	20.33	33.46			
Hospital region			0.7738	3	0.5087
Northeast	14.23	17.61			
Midwest	26.83	25.48			
South	36.59	36.89			
West	22.36	20.02			
Hospital location			1.541	2	0.2146
Rural	4.88	7.5			
Urban nonteaching	19.51	19.84			
Urban teaching	75.61	72.66			
Mortality			4.6987	1	0.0304
No	91.06	95.16			
Yes	8.94	4.84			
Admission type			1.291	1	0.2561
Elective	17.14	20.11			
Non-elective	82.86	79.89			
Length of stay			8.0136	1	0.0047
5 days or less	50	59.58			
More than 5 days	50	40.42			
Charlson Comorbidity Index			1.1812	2	0.3073
0 – 4	63.82	68.69			
5 – 7	21.14	17.36			
⩾ 8	15.04	13.95			

Table 4 shows the risk factors for arrhythmias, demonstrating that older age (≥ 65 years) was strongly associated with increased odds of arrhythmias (AOR 2.20, 1.49-3.25). Female patients exhibited a lower likelihood of developing arrhythmias (AOR: 0.61, 0.46-0.81). This gender difference may be influenced by hormonal, electrophysiological, or diagnostic differences, as women are known to present differently or may be underdiagnosed with arrhythmias. Additionally, longer hospital stays (>5 days) remained a significant predictor of arrhythmia (AOR: 1.37, 1.03-1.83). This suggests that arrhythmia could be developed as a complication of prolonged hospitalization due to stress, metabolic imbalances, or medication effects, or that patients with arrhythmia require longer hospitalization due to greater monitoring needs. Other factors, such as race/ethnicity, median household income, insurance type, hospital region, hospital location, mortality, admission type, and the Charlson Comorbidity Index, did not exhibit an association with arrhythmia. Although the association between mortality and arrhythmia (AOR 1.56, 0.90-2.70) did not reach statistical significance, the p-value was approaching significance, indicating a possible trend toward increased mortality in this group.

**Table 4:** Factors Associated With Arrhythmias Among Hospitalized Patients With Carcinoid Syndrome. OR = odds ratio; CI = confidence interval The models were adjusted for age, race/ethnicity, gender, hospital region, hospital location, Charlson comorbidity index, insurance, length of stay, mortality, type of admission, and median household income national quartiles. Race/ethnicity categories follow the NIS database classification. "Other" includes Hispanics, Asian or Pacific Islander, Native American, and other racial groups per NIS definitions. Hispanic, Asian or Pacific Islander, Native American and other racial individuals were included in the "Other" category due to a sample size below the reporting threshold (n=10) per NIS database recommendations. * Indicates statistical significance of p<0.05.

Characteristics	OR (95% CI)	
	Unadjusted	Adjusted
Age		
Less than 65	Reference	Reference
65 years and above	2.36 (1.75–3.19)*	2.20 (1.49–3.25)*
Gender		
Male	Reference	Reference
Female	0.58 (0.44–0.77)*	0.61 (0.46–0.81)*
Race/ethnicity		
Non-Hispanic White	Reference	Reference
Non-Hispanic Black	0.79 (0.48–1.27)	0.85 (0.50–1.44)
Other	0.68 (0.40–1.16)	0.64 (0.36–1.14)
Median household income national quartiles		
Quartile 1	Reference	Reference
Quartile 2	1.21 (0.79–1.83)	1.17 (0.76–1.80)
Quartile 3	1.29 (0.85–1.96)	1.17 (0.74–1.85)
Quartile 4	1.36 (0.90–2.06)	1.39 (0.88–2.21)
Insurance type		
Medicare	Reference	Reference
Medicaid	0.79 (0.46–1.35)	1.55 (0.81–2.99)
Private/Other	0.49 (0.35–0.69)*	0.86 (0.56–1.30)
Hospital region		
Northeast	Reference	Reference
Midwest	1.30 (0.83–2.04)	1.39 (0.86–2.26)
South	1.23 (0.81–1.85)	1.30 (0.84–2.03)
West	1.38 (0.88–2.17)	1.46 (0.91–2.36)
Hospital location		
Rural	Reference	Reference
Urban nonteaching	1.51 (0.78–2.93)	1.29 (0.64–2.58)
Urban teaching	1.60 (0.87–2.94)	1.57 (0.82–3.00)
Mortality		
No	Reference	Reference
Yes	1.93 (1.18–3.15)*	1.56 (0.90–2.70)
Admission type		
Non-elective	Reference	Reference
Elective	0.82 (0.58–1.17)	0.87 (0.60–1.28)
Length of stay		
5 days or less	Reference	Reference
More than 5 days	1.47 (1.13–1.92)*	1.37 (1.03–1.83)*
Charlson Comorbidity Index		
0 – 4	Reference	Reference
5 – 7	1.31 (0.93–1.85)	1.24 (0.86–1.78)
⩾ 8	1.16 (0.77–1.75)	0.96 (0.63–1.47)

Several comorbidities were significantly associated with CHF and arrhythmia (Table 5). Our results demonstrated that acute myocardial infarction (AOR 2.99, 1.93-4.63), chronic obstructive pulmonary disease (COPD) (AOR 1.88, 1.39-2.54), as well as peripheral vascular disease (AOR: 1.57, 1.01-2.46), were strong predictors of CHF. Renal disease also significantly increased the odds of CHF (AOR 2.61, 1.95-3.49). Similarly, acute myocardial infarction (AOR: 1.90, 1.09-3.30), renal disease (AOR: 1.51, 1.08-2.11), and COPD (AOR: 1.57, 1.12-2.20) were significant predictors of arrhythmias. Notably, the AORs for CHF were higher than those for arrhythmia, particularly for acute myocardial infarction and renal disease, suggesting that these conditions have a stronger predictive value for CHF than for arrhythmia. This may reflect that CHF itself could be a mediating factor in the relationship between these conditions and arrhythmia. Other comorbidities, including cerebrovascular disease, dementia, rheumatoid disease, diabetes mellitus, and metastatic cancer, did not show an association with CHF and arrhythmia. Interestingly, Diabetes mellitus did not show an association with either outcome, which is unexpected given that diabetes is a known risk factor for both conditions. The effects of diabetes may have already been captured through its complications, such as peripheral vascular disease and renal disease. Also, dementia and cerebrovascular disease had AORs close to 1 with wide CIs, indicating no significant association with CHF or arrhythmia, however, literature suggests that stroke and cognitive impairment are often linked to arrhythmia, raising the possibility of underreporting or a lack of major impact in this specific cohort. Table 6 summarizes the main findings and predictors for CHF and arrhythmias in hospitalized patients with CS.

**Table 5:** Comorbidities That Predict Congestive Heart Failure and Arrhythmias Among Hospitalized Patients With Carcinoid Syndrome. AOR = adjusted odds ratio; CI = confidence interval The models were adjusted for acute myocardial infarction, peripheral vascular disease, cerebrovascular disease, dementia, chronic obstructive pulmonary disease (COPD), rheumatoid disease, diabetes mellitus, renal disease, and metastatic cancer. * Indicates statistical significance of p<0.05.

Comorbidities	AOR (95% CI)	
	Congestive Heart Failure	Arrhythmias
Acute myocardial infarction	2.99 (1.93–4.63)*	1.90 (1.09–3.30)*
Peripheral vascular disease	1.57 (1.01–2.46)*	1.14 (0.68–1.90)
Cerebrovascular disease	0.96 (0.51–1.79)	1.09 (0.52–2.29)
Dementia	1.20 (0.64–2.25)	0.53 (0.22–1.25)
COPD	1.88 (1.39–2.54)*	1.57 (1.12–2.20)*
Rheumatoid disease	1.13 (0.61–2.12)	0.65 (0.26–1.62)
Diabetes mellitus	0.98 (0.72–1.35)	0.85 (0.59–1.23)
Renal disease	2.61 (1.95–3.49)*	1.51 (1.08–2.11)*
Metastatic cancer	0.98 (0.72–1.33)	0.82 (0.57–1.16)

**Table 6:** Summary of Significant Predictors for CHF and Arrhythmias Among Hospitalized Patients With Carcinoid Syndrome AOR = adjusted odds ratio; CI = confidence interval; CHF =  congestive heart failure * Indicates statistical significance (p < 0.05).

Predictors	CHF (AOR, 95% CI)	Arrhythmia (AOR, 95% CI)	Interpretation
Age ≥ 65 years	1.23 (0.89–1.69)	2.20 (1.49–3.25)*	Significant predictor for arrhythmia but not for CHF.
Gender (female)	0.79 (0.61–1.01)	0.61 (0.46–0.81)*	Significant predictor for arrhythmia but not for CHF.
Non-Hispanic Black	2.05 (1.43–2.94)*	0.85 (0.50–1.44)	Significant predictor for CHF but not for arrhythmia.
Non-Hispanic other	2.07 (1.18–3.65)*	0.64 (0.36–1.14)	Significant predictor for CHF but not for arrhythmia.
Medicare	0.54 (0.28–1.01)	1.55 (0.81–2.99)	No significant association for both CHF and arrhythmia.
Private insurance	0.65 (0.46–0.93)*	0.86 (0.56–1.30)	Significant predictor for CHF but not for arrhythmia.
Hospital region	No significant association	No significant association	No regional disparities for CHF or arrhythmia.
Hospital location	No significant association	No significant association	No location-based disparities for CHF or arrhythmia.
Length of stay > 5 days	1.35 (1.05–1.73)*	1.37 (1.03–1.83)*	Significant predictor for both CHF and arrhythmia.
Mortality	1.67 (1.05–2.66)*	1.56 (0.90–2.70)	Significant predictor for CHF but not for arrhythmia.
Acute myocardial infarction	2.99 (1.93–4.63)*	1.90 (1.09–3.30)*	Significant predictor for both CHF and arrhythmia.
Peripheral vascular disease	1.57 (1.01–2.46)*	1.14 (0.68–1.90)	Significant predictor for CHF but not for arrhythmia.
Renal disease	2.61 (1.95–3.49)*	1.51 (1.08–2.11)*	Significant predictor for both CHF and arrhythmia.
COPD	1.88 (1.39–2.54)*	1.57 (1.12–2.20)*	Significant predictors for both CHF and arrhythmia.
Dementia	1.20 (0.64–2.25)	0.53 (0.22–1.25)	No significant association for both CHF and arrhythmia.
Cerebrovascular disease	0.96 (0.51–1.79)	1.09 (0.52–2.29)	No significant association for both CHF and arrhythmia.
Diabetes mellitus	0.98 (0.72–1.35)	0.85 (0.59–1.23)	No significant association for both CHF and arrhythmia.

## Discussion

NETs are neoplasms that usually grow slowly over the years, commonly causing few or no symptoms until they are large or have metastasized [31]. Heart disease frequently occurs in patients with CS and is responsible for substantial morbidity and mortality [33]. Hence, it is a reasonable strategy to investigate the factors associated with mortality among hospitalized patients with CS, with a specific focus on individuals with CHF and arrhythmia. Our data shows that CHF and arrhythmias are common complications in patients with CS, with significant clinical and demographic predictors that could enhance risk stratification and management strategies.

Aged-related heart disease causes progressive changes in the heart leading to a higher prevalence of CHF and arrhythmia [34, 35]. Our results demonstrated that increasing age was significantly associated with CHF and arrhythmias, with the majority of affected patients being 65 years or older. This aligns with previous studies indicating that age-related cardiovascular remodeling is compounded by chronic exposure to serotonin [8, 16, 36-39]. Serotonin has been shown to induce pathological remodeling of hearts through different mechanisms. Experiments with exposure to serotonin have induced heart diseases in animal models [8, 36]. In addition, the animals developed CS due to an increased number of interstitial cells and extracellular matrix synthesis [8]. Also, serotonin has been linked to the renin-angiotensin system, promoting inflammation, cardiac hypertrophy, collagen synthesis, oxidative stress, and valve thickening in mice and humans [37-39]. Moreover, elevated levels of serotonin can stimulate 5-HT4 receptors leading to focal atrial extrasystoles due to calcium overload in the cardiac myocytes, consequently causing delayed depolarization and shortening of the atrial effective refractory period [16]. However, it remains unclear whether serotonin acts as an independent driver of pathology or exacerbates pre-existing age-related cardiac remodeling. Additionally, our data showed that male patients exhibited a higher prevalence of CHF and arrhythmia, suggesting possible sex-related differences in susceptibility. Studies have shown that individuals of different sexes and genders differ regarding the prevalence and management of CHF and arrhythmia [40, 41]. The higher prevalence of CHF and arrhythmia among male patients could reflect broader epidemiological trends in the general population, where men exhibit higher susceptibility to cardiac dysfunction. These discrepancies may be related to sex hormones, sex differences in serotonin metabolism, receptor expression, and other factors; however, studies are necessary to fully understand the molecular mechanisms related to sex, gender, and heart disease.

Racial and socioeconomic factors lead to disparities in the diagnosis, management, as well as outcomes [42]. Race and ethnicity emerged as independent predictors of CHF, with non-Hispanic Black and non-Hispanic/other races demonstrating increased odds. It has been shown that there is a significant disparity in cardiovascular disease risk factors across various racial and ethnic groups [43]. The socioeconomic disparities, such as differences in access to healthcare, diagnostic resources, and early intervention, may contribute to the observed racial disparities in CHF risk [44]. Also, genetic factors influencing myocardial structure and function may further underline these racial differences [45, 46]. These differences include worse arterial stiffness and microcirculatory function [45], lower levels of natriuretic peptides [47], and differences in the left ventricular structural and functional changes in response to arterial afterload [46]. Structural, functional, and vascular differences, or responses to therapies, may also potentially explain the higher incidence of CHF and worse outcomes for the non-Hispanic Black population [42]. Interestingly, our data demonstrated that private insurance was associated with a reduced risk of CHF, potentially reflecting variations in healthcare utilization, access to specialized care, and earlier intervention. The association between private insurance and reduced CHF risk suggests differences in early intervention and access to care, which may reflect selection bias, as healthier individuals are more likely to maintain private insurance.

Prolonged hospital stays were significantly associated with cardiovascular diseases, showing the burden of these complications on healthcare resources and patient outcomes [48]. The higher CCI among CHF patients further emphasizes the impact of multimorbidity in this population. Particularly, we found that CS was significantly associated with different comorbidities, including acute myocardial infarction, renal disease, COPD, and peripheral vascular disease, which increased the probability of CHF and arrhythmias. Renal disease may exacerbate CHF through different mechanisms including volume overload, electrolyte imbalances, and increased vascular resistance [49, 50]. COPD may contribute to arrhythmia through hypoxia-induced autonomic dysfunction [51, 52]. Peripheral vascular disease may serve as a marker of systemic atherosclerosis and endothelial dysfunction, contributing to hemodynamic stress and increased cardiac workload [53, 54]. These findings emphasize the interplay between systemic conditions and carcinoid-related cardiovascular dysfunction, reinforcing the need for a multidisciplinary approach in patient management.

However, our study has limitations. Data obtained from publicly available databases is at risk from errors in procedure coding or incorrect diagnostic labeling. Although in-hospital mortality could be assessed, distinguishing specific causes of death could not be appropriately differentiated. Additionally, the absence of a present-on-admission indicator prevents differentiation between pre-existing conditions and those diagnosed during hospitalization [55]. Moreover, the dataset does not include detailed clinical variables, such as serotonin levels and echocardiographic parameters, which could offer valuable mechanistic insights. However, the strengths of this study include the use of a large, nationally representative dataset, which enhances the generalizability of our findings. Additionally, the multivariate analysis allowed for the identification of independent predictors.

## Conclusions

This study identified key predictors of CHF and arrhythmias among hospitalized patients with CS. Our results emphasize the importance of a multidisciplinary approach in managing CS patients with CHF and arrhythmia to improve care delivery and reduce mortality. Collaboration among specialists in cardiology, oncology, and endocrinology is essential for comprehensive patient management, as it can facilitate earlier detection of CHF and arrhythmias, improve serotonin management, and enhance surgical outcomes for valve disease. The findings underscore the importance of early risk stratification and targeted interventions to mitigate cardiac complications in CS patients. Additionally, incorporating our data into clinical practice has the potential to improve early recognition, promoting timely interventions for CS patients with CFH and arrhythmia. Future research should focus on integrating clinical scoring systems, biomarkers (natriuretic peptides, serotonin levels, and galectin-3), and echocardiographic findings to refine the understanding of pathophysiology and improve patient management in this complex condition.
